# The efficacy of apneic oxygenation during intubation using a prototype of an oxygenation laryngoscope - a technical simulation

**DOI:** 10.1186/s12871-023-02234-6

**Published:** 2023-08-14

**Authors:** Wolfgang A Wetsch, Daniel C Schroeder, Susanne J Herff, Bernd W Böttiger, Volker Wenzel, Holger Herff

**Affiliations:** 1 Faculty of Medicine, University of Cologne, Albertus-Magnus-Platz 1, 50931 Cologne, Germany; 2 Department of Anaesthesiology and Intensive Care Medicine, University Hospital of Cologne, Cologne, Germany; 3 Department of Anaesthesiology and Intensive Care, German Armed Forces Central Hospital, Koblenz, Germany; 4 Department of Anesthesiology, Friedrichshafen Regional Medical Center, Friedrichshafen, Germany; 5 Department of Anesthesiology, University of Florida, Gainesville, FL USA; 6 Department of Anesthesiology, PAN Clinic, Cologne, Germany

**Keywords:** Laryngoscope, Manikins, Oxygen, Insufflation, Trachea, Apnea, Intubation, Intratracheal

## Abstract

**Background:**

Recently, a non-commercial oxygenation laryngoscope was able to maintain apneic oxygenation during simulated intubation efforts. Since that prototype was 3 mm wider than a standard Macintosh laryngoscope blade, the intubation performance of this device may differ from standard blades. A new prototype of an oxygenation laryngoscope was developed, consisting of a standard-size Macintosh blade and a fixed oxygen supply line to the side. Actually, it is unclear at which point of this blade the oxygen supply line should end to facilitate the best possible oxygen supply for apneic oxygenation.

**Methods:**

In this simulation study using a standardized human airway manikin, the efficacy of apneic oxygenation by oxygen insufflation using standard and modified Macintosh blades was compared: a standard Macintosh blade without oxygen supply line as control, one with an additional oxygen supply line ending proximal near the handle, one with the line ending at the middle of the blade, and one with the line ending near the tip. A preoxygenated test lung was connected to an oximeter with a flow rate of 200ml/min, simulating oxygen consumption of a male adult, and to the trachea of an anatomically correctly shaped airway manikin. Apneic oxygenation was performed and oxygen content was measured over a 20-minutes observation period. Experiments were repeated five times for each laryngoscope blade.

**Results:**

Oxygen percentage in the test lung dropped from 100 ± 0% at the start of the experiment to 53 ± 1.5% in the room air control group (p < 0.001 compared to all other groups), and to 74 ± 2.5% in the proximal oxygen line group, whereas oxygen percentage remained at 100% in both the medium and distal oxygen line groups (p = 1 between these groups; p < 0.001 between all other groups).

**Conclusions:**

In this simulation study with a preoxygenated airway manikin, the use of a modified Macintosh laryngoscope blade with oxygen line attached at the tip or at the middle were able to maintain apneic oxygenation without measurable drop of oxygen content over 20 min. Proximal placement of the oxygen supply line still showed an advantage against room air, however it did not completely prevent room air from entering the airway.

**Trial registration:**

Not applicable.

## Background

Before planned airway management in anesthesia, critical care and emergency medicine, preoxygenation and denitrogenization of the patient’s lungs is considered mandatory, as it allows the provider a longer time for airway management before oxygen saturation falls and hypoxemia occurs. The time until oxygen desaturation occurs during intubation efforts can be even prolonged by applying the technique of apneic oxygenation [1, 2]. Several years ago, a non-commercial oxygenation laryngoscope was developed and evaluated for that purpose [3, 4]. However, due to its specific construction – which was done by welding two standard Macintosh laryngoscope blades on top of each other, thus generating a channel that allows insufflated oxygen to reach the tip – the prototype was 3 mm wider and higher than a standard Macintosh laryngoscope blade. This wider laryngoscope blade also required a special handle to fit it, and its modification has also altered the shape of the laryngoscope itself, thus this may have the potential to make intubation more difficult when using this blade, especially in more challenging airways. Furthermore, due to its construction, cross-contamination and hygiene concerns have prevented evaluation of that blade in humans so far.

In the need for a standard-sized blade with the possibility of providing oxygen for apneic oxygenation, we designed a new prototype by using a disposable standard Macintosh blade and adding an additional oxygen supply line. The old oxygenation laryngoscope always insufflated oxygen at the most distal point – which is most difficult to achieve. So far, it is not known if this is necessary at all, or if the oxygen outlet could also be placed more proximally to achieve the same effect on oxygenation. Thus, we constructed three different new oxygenation laryngoscopes with standard Macintosh blades with different lengths of the oxygen supply line. In an established bench model of apneic oxygenation with a preoxygenated test lung attached to a manikin, [3, 4] we measured decreases in oxygen percentage for room air as a control and while applying oxygen with all three prototype laryngoscope blades. We hypothesized that there would be no difference in oxygen fraction decrease between groups.

## Methods

### Ethics approval

Since this is a technical simulation without involvement of volunteers or patients, involvement of the Ethics Committee is not required based in national regulations.

### Experimental setup

**Fig. 1:**
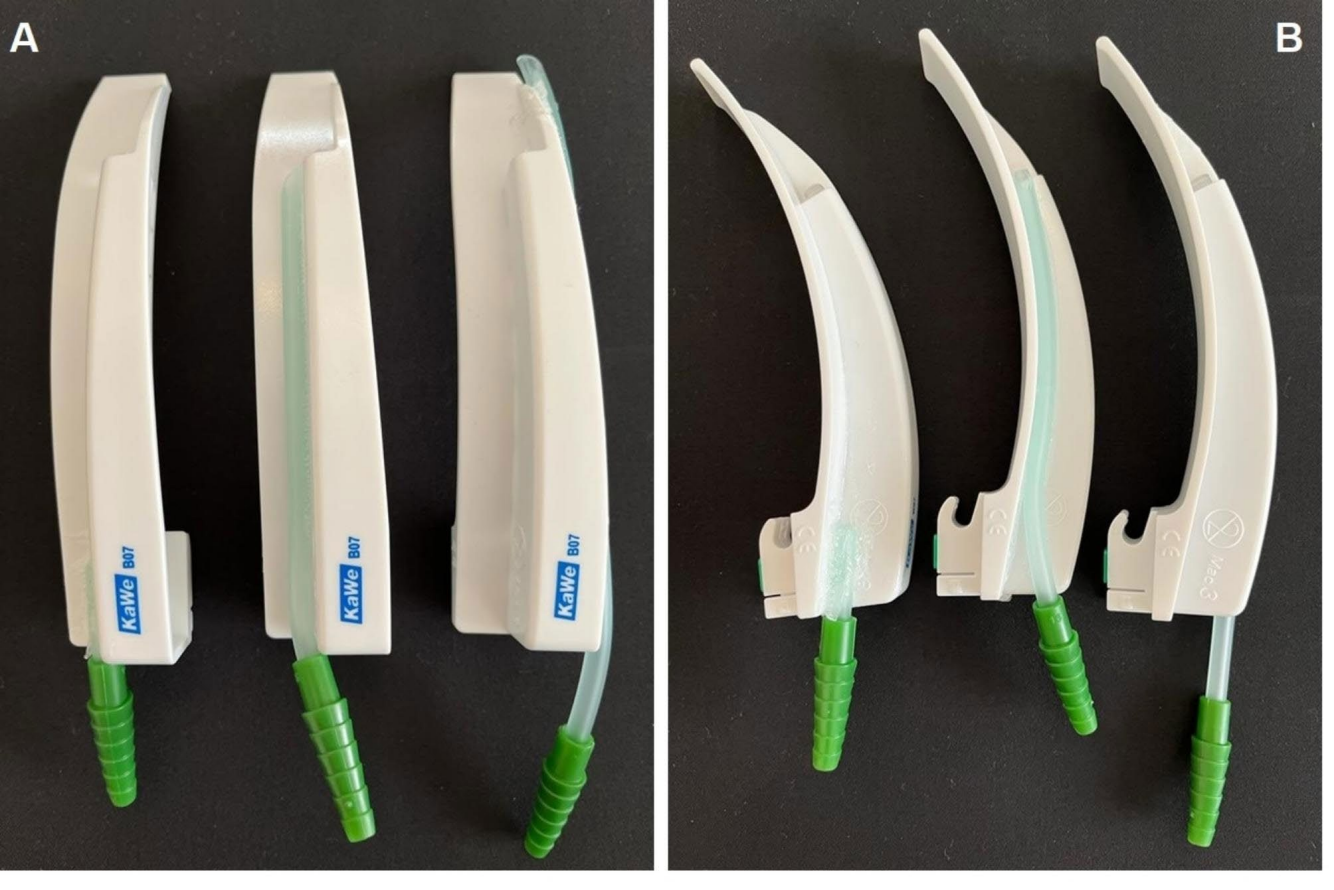
Photos of all laryngoscope blades with short, medium and long oxygen supply line from top view (**A**) and side view (**B**). Note the standard size of Macintosh blades

Three different new oxygenation laryngoscopes with standard Macintosh blade were constructed: the first one with the oxygen supply line ending proximal near the handle on the interior side, the second one with the line ending at the height of the light source on the right side and a third one with the line ending distally; due to construction reasons we had to fix this line on the left side. (Fig. 1) The trachea of a male intubation manikin (AirSim Combo Bronchi X, TruCorp, Lurgan, Northern Ireland) was connected to a test lung with a capacity of 3 L, representing the functional residual capacity (FRC) of an adult man. Subsequently, an oximeter with probe suction rate of 200 mL/min (Datex Ohmeda Cardiocap 5, GE Healthcare, Chalfont St Giles, UK) was connected at the base of the test lung. The sample volume was not given back to the test lung; thus, the drawn gas volume equals the amount of oxygen that would be consumed in an adult human during apneic oxygenation [5].

### Experimental procedure

**Fig. 2:**
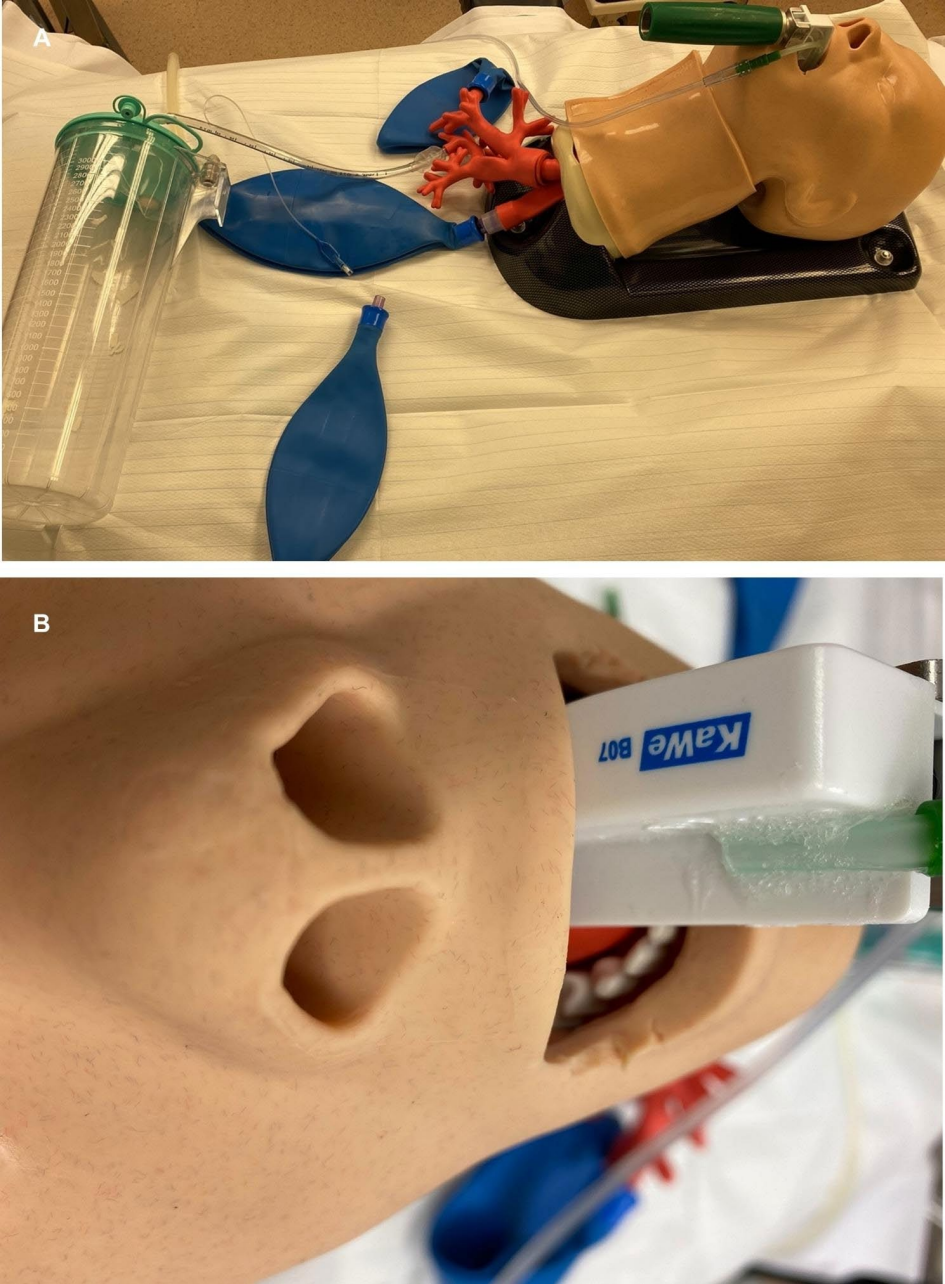
Experimental setting (**A**) with placement of the laryngoscope blade with a short oxygen supply line (**B**)

Prior to each experiment, the test lung was filled with 100% oxygen until that value was achieved on the monitor for at least one minute. 3 L/min oxygen were insufflated via the oxygen supply line of all three prototype laryngoscope blades, whereas no oxygen was insufflated in the control group. In random order, the laryngoscopes were put in typical position for intubation with the handle stabilizing the whole experimental setting (Fig. 2). Five experiments were performed for each group, measuring the decrease in oxygen percentage for 20 min, starting from 100% at baseline.

### Statistical evaluation

Data is reported as mean plus / minus standard deviation. After Kolmogorov Smirnov analysis, post hoc analysis of variances (ANOVA) was performed to determine overall statistical significance between the groups; to assess differences between the groups, Student Newman Keuls test was performed. A *P* value < 0.05 was considered being significant. For both analysis and graph, Sigmaplot 14.0 (Systat Software, San Jose, CA) was used.

## Results

**Fig. 3:**
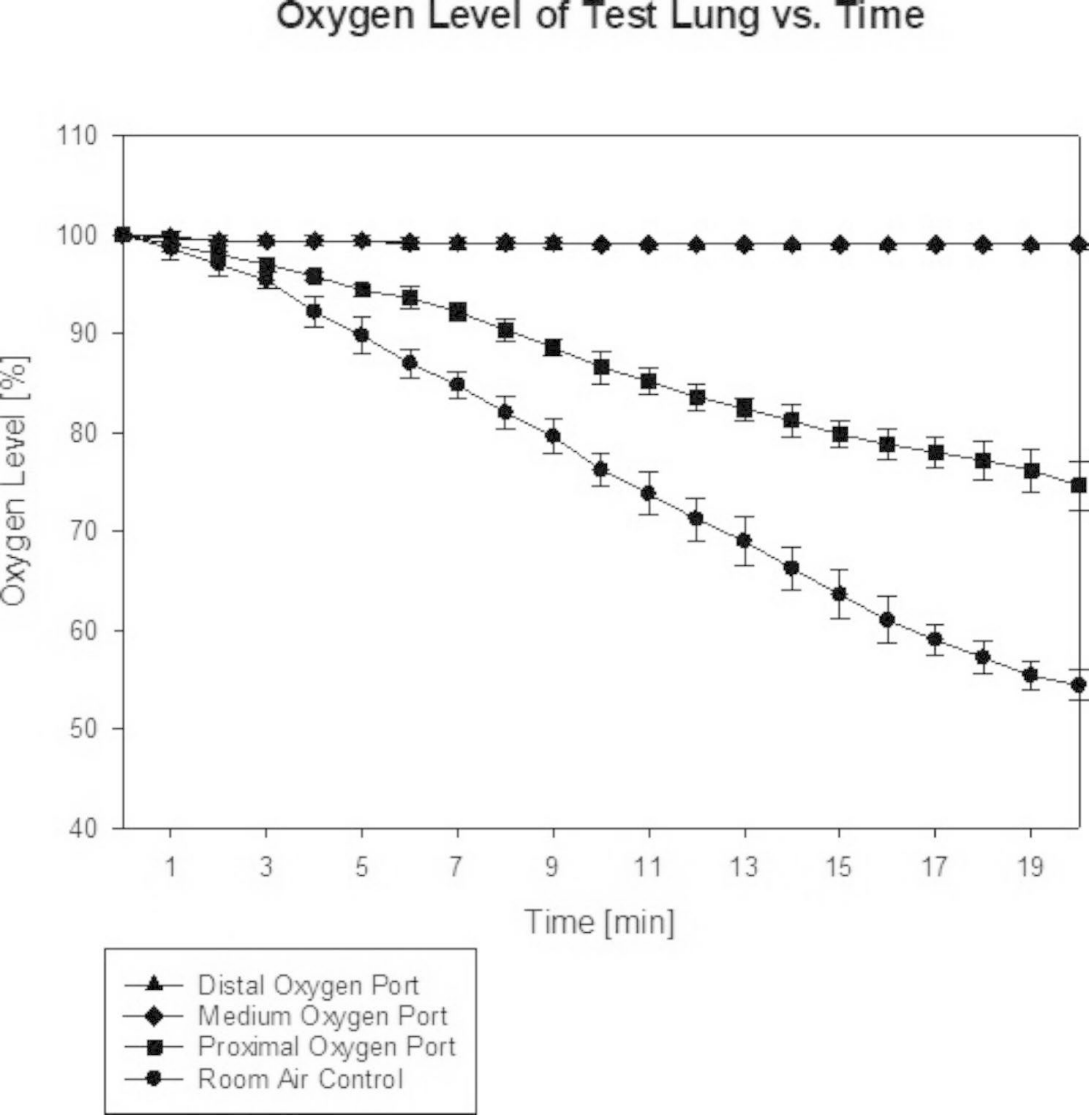
Desaturation of oxygen content in the test lung for the observation period of 20 min

In the room air control group, oxygen percentage in the test lung dropped from 100 ± 0% to 53 ± 2% (p < 0.001 compared to all other groups), and to 74 ± 3% in the short oxygen supply line group, whereas oxygen percentage remained at 100% in both the medium and long oxygen supply line groups (p = 1 between these two groups; between all other groups p < 0.001; Fig. 3).

## Discussion

In this model of apneic oxygenation using different prototypes of oxygenation laryngoscopes, both the distal and medium position of the oxygen supply line were highly efficient in maintaining a 100% oxygen content in the test lungs. The main goal of this model was to assess the ability of the new oxygenation laryngoscope prototypes to facilitate apneic oxygenation using different positions of the oxygen outlet; more proximal outlet positions seem to be easier to build, and might be better for handling of the laryngoscope itself.

To evaluate our hypothesis, a realistic model of the anatomy of the human airway was necessary. In this regard, our anatomically correctly shaped manikin was reasonably reliable to achieve this purpose. We deliberately did not simulate a real apneic oxygenation scenario which either would have meant a human study or at least an animal experiment in a hybrid model connected to a human airway manikin. Our prototype is not a registered medical device and must not be used in humans; furthermore, the anatomy of a usual animal model is completely different from the human airway, so results from animal airway model studies cannot be transferred 1:1 to humans. The prerequisite of continuous denitrogenization of a preoxygenated airway to maintain apneic oxygenation is known for a long time, and our goal was to simulate the ability of different oxygenation laryngoscope prototypes in an anatomically realistic model [6–8]. Thus, we deemed our model suitable for simulating oxygen consumption without using living organisms and that we could spare animal lives by using the stream of 200mL/min of the oxygen meter instead to address the hypothesis of this study.

The main finding of this study is that for optimal continuous denitrogenization of the airway, the prototypes of the oxygenation laryngoscope with both distal and medium oxygen outlet were highly effective in maintaining apneic oxygenation, whereas a proximal outlet was inferior. Interestingly, maintaining denitrogenization was also possible by using very moderate oxygen flows of only 3 L/min. In theory, this relatively low flow should be more than enough, since an oxygen flow of 3 L/min exceeds oxygen consumption of 200mL/min by the factor 15. Controversely, using higher flow rates, we observed in the past that choosing classical high-flow rates even had adverse effects, as this leads to mixing effects with ambient air, most probably caused by venturi effects of the turbulent flow that these high flow rates created [9, 10]. Furthermore, higher gas flows are also known to have negative effects as they may induce direct pressure-related tissue damage and mucosal inflammation [11]. Thus, this study emphasizes again that the optimal point of oxygen insufflation may be an important factor to maintain apneic oxygenation. In this regard, oxygenation laryngoscopes seem to be promising, since they automatically direct the oxygen where it is needed, without the risk of gas mixtures or drawing ambient air into the lungs [11–14]. (Fig. 2).

The possibility of supplying oxygen via a laryngoscope is not entirely new and has been described by others. Using a Miller-shaped blade in neonates has shown to decrease the incidence of severe desaturation during neonatal and infant intubations in a study by Dias et al. [15] However, the use of Miller-shaped blades outside the neonatological and pediatric setting is extremely rare. Since the Macintosh blade type is standard and the by far most widespread laryngoscope type in Europe, we decided to create a modified Macintosh blade. Laryngeal oxygen insufflation has also shown to increase the time to 1% desaturation and reduce the overall rate of desaturation during laryngoscopy in children in a study by Steiner et al.[16]. However, this group used a standard laryngoscope blade to which a tracheal tube was taped with sterile dressing. Unfortunately, their manuscript does not provide photos or an exact description where the tip was placed, so the reproducibility of their experiments is limited. Furthermore, legal restraints would not allow the use of such a self-made device in most countries. Windpassinger et al. [17] used an Airtraq and placed a tracheal tube into the tube guidance channel, which was either used for supplying pharyngeal oxygen or not. The found that supplying oxygen prolonged the time to desaturation. Again, the study was performed in infants and young children, and the tip position was not standardized, so it may have included various positions where the tube had ended, and the optimum position remains unclear. However, the importance of the position has been identified in several publications [18].

From what is known about the few commercially available oxygenation laryngoscopes, based on pictures from the manufacturers, we hypothesize that the oxygen ports of these devices are ending at the medium or proximal end of the laryngoscope blades [19, 20]. In this regard, it could be discussed based on our results if a deeper point of oxygen supply on the blade could improve these devices as well and should be subject of further studies. A more distal position may be helpful in regard of blocking room air from entering the airway. A more proximal ending of the oxygen supply may be less effective in preventing room air from entering into the airway which reduces efficiency of apneic oxygenation but may improve sight on the glottis. In our model, we were not able to decide whether a medium or distal supply point is more effective, as the difference between these two points may have been too small to detect a difference.

This version of the oxygenation laryngoscope may have some theoretical advantages over the previous version concerning the handling, since the shape of the conventional Macintosh blade itself has not been altered and only an oxygen line was added; the oxygen port might be integrated into the blade in a future version so that the handling remains exactly the same. Furthermore, the use of a disposable blade in our experiments has theoretical advantages over the reusable prototype used in previous studies, since cross-contamination of infectious pathogens cannot occur any more. In a next step, to prove these assumptions, it may be promising to assess handling quality of both concepts.

### Limitations

Several limitations of this study must be mentioned. First, as the device is a self-made, modified Macintosh laryngoscope blade and not a registered and approved medical device, the study had to be conducted as a simulation trial in a manikin model, as legislation in the European Union prohibits the use in humans. Nevertheless, this model was deemed suitable for determining the most efficient design variant of the oxygenation laryngoscope from previous trials. Second, this model measured oxygen percentage; data from this model cannot be used to directly estimate times until oxygenation deteriorates. Third, we have not evaluated whether the changes may affect the performance of the laryngoscope in a difficult airway. Fourth, using laryngoscopes as devices for apneic oxygenation only works when attempting intubation with inserted laryngoscopes, and does not replace alternative ways of apneic oxygenation when different approaches are chosen.

## Conclusions

In this simulation study with a preoxygenated airway manikin, the use of a modified Macintosh laryngoscope blade with oxygen line attached at the tip or at the middle were able to maintain apneic oxygenation without measurable drop of oxygen content over 20 min. Proximal placement of the oxygen supply line still showed an advantage in comparison with room air, however it did not completely prevent room air from entering the airway.

## Data Availability

All data generated used or analyzed during this study are included in the published article.
